# MEKK1-Dependent Activation of the CRL4 Complex Is Important for DNA Damage-Induced Degradation of p21 and DDB2 and Cell Survival

**DOI:** 10.1128/MCB.00081-21

**Published:** 2021-09-24

**Authors:** Susanne Bacher, Hilda Stekman, Carla M. Farah, Annika Karger, Michael Kracht, M. Lienhard Schmitz

**Affiliations:** a Institute of Biochemistry, Justus Liebig University, Member of the German Center for Lung Research, Giessen, Germany; b Rudolf Buchheim Institute of Pharmacology, Justus Liebig University, Member of the German Center for Lung Research, Giessen, Germany

**Keywords:** cullin-4, ubiquitination, MEKK1, DNA damage, cell cycle, apoptosis, mitogen-activated protein kinases

## Abstract

Cullin-4 ubiquitin ligase (CRL4) complexes are differentially composed and highly dynamic protein assemblies that control many biological processes, including the global genome nucleotide excision repair (GG-NER) pathway. Here, we identified the kinase mitogen-activated protein kinase kinase kinase 1 (MEKK1) as a novel constitutive interactor of a cytosolic CRL4 complex that disassembles after DNA damage due to the caspase-mediated cleavage of MEKK1. The kinase activity of MEKK1 was important to trigger autoubiquitination of the CRL4 complex by K48- and K63-linked ubiquitin chains. MEKK1 knockdown prohibited DNA damage-induced degradation of the CRL4 component DNA-damage binding protein 2 (DDB2) and the CRL4 substrate p21 and also cell recovery and survival. A ubiquitin replacement strategy revealed a contribution of K63-branched ubiquitin chains for DNA damage-induced DDB2/p21 decay, cell cycle regulation, and cell survival. These data might also have implications for cancer, as frequently occurring mutations of MEKK1 might have an impact on genome stability and the therapeutic efficacy of CRL4-dependent immunomodulatory drugs such as thalidomide derivatives.

## INTRODUCTION

Cullin-RING ligases (CRLs) are the largest family of ubiquitin E3 ligases and are composed of modular protein subunits (1). The human genome encodes seven cullin proteins which function as scaffolding proteins and associate with a RING finger protein (RING of cullins [ROC1/RBX1 or ROC2/RBX2]) to recruit the E2 ubiquitin-conjugating enzyme (2). The N-terminal part of cullins binds to a number of specific adapter proteins that, in turn, bind to substrate receptors. Cullin-4 (CUL4A and the highly related protein CUL4B) typically use the DNA-damage binding protein 1 (DDB1) adaptor to interact with a variety of DDB1–cullin-4-associated factors (DCAFs) that make contact to substrate proteins or that can be also ubiquitinated and degraded by themselves (3). Activation of the CRL4 complex needs the covalent but reversible ligation of the ubiquitin-like protein NEDD8 (neural precursor cell-expressed developmentally downregulated 8) to a conserved lysine in the C terminus of the cullin (4). The CRL4 complex is involved in the regulation of several processes, including transcription and embryonic development (5, 6). This complex also participates in the multistep global genome nucleotide excision repair (GG-NER) pathway, where a heterodimer of DDB1 and DDB2 recognizes photolesions in the DNA to promote several consecutive steps in the early phase of DNA repair (3, 7). While CRL4-mediated monoubiquitination of histone proteins leads to their eviction around the damaged site, DDB1/DDB2 allow the subsequent recruitment of the xeroderma pigmentosum complementation group C (XPC) DNA repair complex. CRL4-mediated ubiquitination of XPC increases its affinity for damaged DNA (8), and later on, CRL4-mediated K48-linked ubiquitination of DDB2 enables access of further NER factors and prevents the proapoptotic function of DDB2 following DNA damage (9). Further CRL4 substrates include regulators of cell proliferation, including proliferating cell nuclear antigen (PCNA), p27, and p21 (10).

All cullin complexes, including CRL4, are highly dynamic and differentially composed assemblies that are regulated at many levels. While the neddylation status is affected by (de)neddylating enzymes, the Cand1/2 (cullin-associated and neddylation-dissociated protein 1/2) proteins act as exchange factors for pools of nonsubstrate-bound adaptor-substrate receptors (11). The CRL4 complex is also regulated by phosphorylations and further posttranslational modifications (6, 12). CRL4 was found to interact in a context-specific manner also with kinases, including oxidative stress-responsive kinase 1 (OSR1), cyclin-dependent kinase 1 (CDK1), inositol phosphate kinase (IP6 kinase), and ataxia telangiectasia and Rad3 related (ATR) (13–16), but their precise function for the regulation of CRL4 assembly and functions is still not clear.

Consistent with its role in fundamental processes, components of CRL4 complexes are frequently overexpressed in a variety of cancers (17). The therapeutical relevance of CRL4 stems from the finding that immunomodulatory drugs such as thalidomide and its derivatives, which are used for the treatment of multiple myeloma, bind to the CRL4 substrate receptor cereblon (18). This interaction alters the substrate specificity of CRL4 and allows the degradation of oncogenic neosubstrates, including casein kinase 1 alpha (CK1α), resulting in anticancer effects (19).

Also, the serine/threonine kinase mitogen-activated protein kinase kinase kinase 1 (MEKK1) is found to be frequently deregulated in cancer, with somatic missense or nonsense mutations and copy number loss frequently occurring in luminal breast cancer (20). MEKK1 belongs to the group of MAP3 kinases and displays a dual function by acting as a kinase and also as a ubiquitin E3 ligase via a plant homology domain (PHD) domain (21). UV irradiation and DNA-damaging chemicals can activate the kinase function of MEKK1 and, later on, induce the proteolytic cleavage of MEKK1 by caspases (22, 23). While the full-length MEKK1 generates antiapoptotic signals, the shorter 91-kDa form generated by caspase-cleavage exerts proapoptotic functions (24, 25). MEKK1 plays a large number of different functions in cell proliferation (26) and cell migration, leading to a defect in eyelid closure in mice lacking the MEKK1-encoding *Map3k1* gene (27). In addition, MEKK1 is also involved in the regulation of rapid signaling events in response to cellular stress (DNA damage, cytokines, and osmotic stress) (28, 29).

We have previously found the association of MEKK1 with the cullin-4 substrate receptor DCAF7 (30) and were thus interested to test whether this kinase can also associate with the CRL4 complex. We found the constitutive association of endogenous MEKK1 with cullin-4 and DDB1 that was lost after prolonged DNA damage upon caspase-mediated cleavage of MEKK1. The kinase function of MEKK1 was required to allow autoubiquitination of the CRL4 complex by K63- and K48-linked ubiquitin chains. MEKK1 was required for cell survival and CRL4-mediated DDB2/p21 decay in cells treated with DNA-damaging agents. A ubiquitin replacement system showed the contribution of K63-branched ubiquitin chains for the survival, DDB2/p21 decay, and cell cycle reentry of cells after induction of DNA damage.

## RESULTS

### MEKK1 associates with the endogenous CRL4 complex.

**FIG 1:**
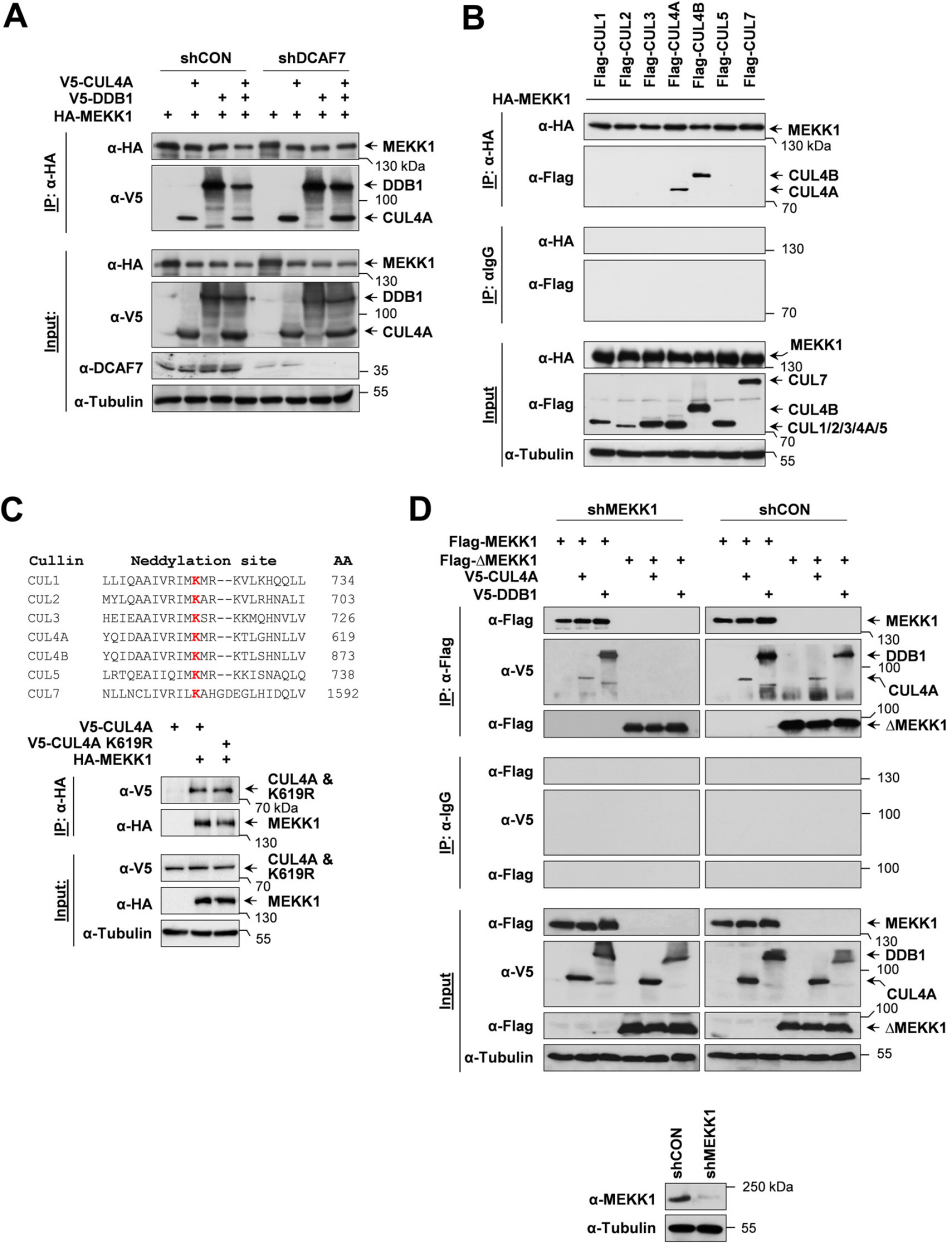
MEKK1 interacts with components of the CRL4 complex. (A) Equal numbers of HEK293 cells were transfected with vectors encoding shRNAs against DCAF7 or a scrambled control (shCON), selected by puromycin treatment for 2 days to eliminate untransfected cells, and then transfected to express MEKK1 and the indicated components of the CRL4 complex. One part of the cell lysates was used for coimmunoprecipitation experiments (IP) as shown; another part was used as input controls to control proper knockdown and protein expression. Tubulin was detected to ensure comparable protein loading. The position of a molecular weight marker is shown. (B) HEK293 cells were transfected to express HA-MEKK1 in combination with Flag-tagged cullins as indicated. Cell lysates were either analyzed for correct protein expression or used for coimmunoprecipitation experiments using anti-HA or isotype-matched control antibodies. (C, top) Neddylated lysines of human cullin proteins are shown in red, and the positions of the neddylated lysines are indicated. (C, bottom) HA-MEKK1 was coexpressed with V5-tagged cullin-4 or a neddylation-defective mutant, followed by a coimmunoprecipitation experiment. (D) HEK293 cells were transfected with vectors directing the expression of MEKK1-specific shRNA or an unspecific control shRNA and selected for 5 days in puromycin to allow for efficient MEKK1 depletion. (D, bottom) One aliquot of the cells was tested by immunoblotting for efficient knockdown of MEKK1. (D, top) Remaining cells were reseeded in dishes and retransfected to express Flag-tagged MEKK1 or ΔMEKK1 along with CRL4 components as shown, followed by coimmunoprecipitation and immunoblotting.

We previously found that MEKK1-dependent signal outputs were modulated by its interaction partner DCAF7, one of the substrate receptors of the CRL4 complex (30). To test whether MEKK1 also interacts with the CRL4 complex, epitope-tagged versions of MEKK1, CUL4A, and DDB1 were expressed in control cells and in cells where DCAF7 was knocked down with a specific short hairpin RNA (shRNA). Coimmunoprecipitation experiments revealed the association of MEKK1 with CUL4A and DDB1, irrespective of the presence of DCAF7 (Fig. 1A). To address the question of whether MEKK1 association is specific to CUL4A or whether it also binds to CUL4B or other members of the cullin family, further coimmunoprecipitation experiments were performed. For this purpose, HEK293 cells were transfected to express hemagglutinin (HA)-tagged MEKK1 in combination with further Flag-tagged members of the cullin family. Immunoprecipitated MEKK1 bound only to CUL4A and CUL4B (Fig. 1B), revealing that this kinase selectively binds to both CUL4 paralogs. Further coimmunoprecipitation experiments revealed that the interaction between MEKK1 and CUL4A also occurred for the neddylation-defective CUL4A K619R mutant (Fig. 1C), revealing that also inactive CRL4 complexes can associate with MEKK1. It was then interesting to test whether the kinase domain of MEKK1 is sufficient to bind the CRL4 complex. Cells were transfected to express CUL4A and DDB1 along with a truncated version of MEKK1 consisting of its kinase domain (ΔMEKK1) and a MEKK1-specific shRNA to avoid potential effects of the endogenous wild-type (WT) kinase. Coimmunoprecipitation experiments showed no association of ΔMEKK1 with the CRL4 complex (Fig. 1D), revealing that the kinase domain is not sufficient for this interaction. Intriguingly, binding of ΔMEKK1 to the CRL4 complex occurred in the presence of the endogenous WT kinase (Fig. 1D), raising the need to investigate the behavior of MEKK1 mutants in the absence of the endogenous kinase which might allow self-association.

**FIG 2:**
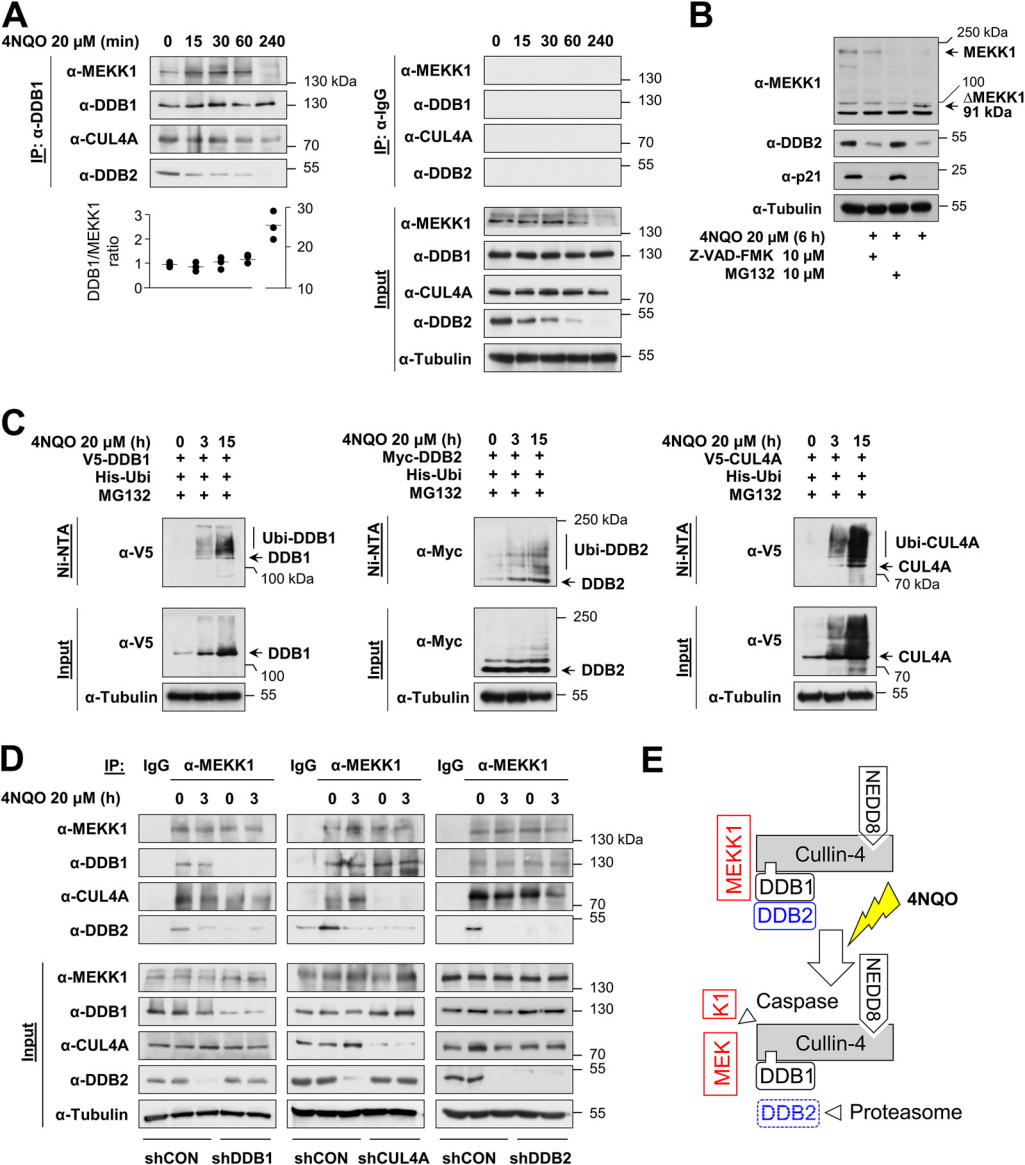
Dynamic regulation of the MEKK1-CRL4 complex in response to DNA damage. (A) HeLa cells were treated with the DNA-damaging agent 4NQO for different time periods, and cell extracts were used for immunoprecipitation with anti-DDB1 or control IgG antibodies; coimmunoprecipitating proteins were detected by immunoblotting. Protein amounts of DDB1 and MEKK1 were quantified using the ChemiDoc imaging system and normalized to tubulin. The DDB1/MEKK1 ratio of untreated controls was set to 1. Values from three independent experiments are shown, and the medians are indicated. (B) HeLa cells were treated with the caspase inhibitor Z-VAD-FMK or the proteasome inhibitor MG132 along with 4NQO for 6 h as shown. Cell extracts were analyzed by immunoblotting. Position of the MEKK1 cleavage product ΔMEKK1 is indicated. (C) HEK293 cells were transfected to express CRL4 components along with His_6_-tagged ubiquitin (His-Ubi). Cells were treated with 4NQO for the indicated periods in the presence of MG132 in order to prevent proteasomal degradation of the ubiquitinated proteins. One fraction of the cells was lysed under denaturing conditions, followed by enrichment of ubiquitinated proteins on Ni-NTA beads and immunoblotting as shown. The position of the ubiquitinated forms of the CRL4 proteins is indicated. (D) HEK293 cells were transfected with vectors encoding shRNAs against DDB1, CUL4A, DDB2, or a scrambled control and selected with puromycin for 2 days, followed by treatment with 4NQO and immunoprecipitation using anti-MEKK1 antibodies. Coimmunoprecipitating proteins were revealed by immunoblotting. (E) Schematic model visualizing the experimental data.

It was then interesting to investigate the MEKK1/CRL4 complex under conditions leading to the activation of CRL4 and also MEKK1, such as DNA damage (31, 32). HeLa cells were treated with the DNA-damaging agent 4-nitroquinoline 1-oxide (4NQO), a UV mimetic agent leading to bulky DNA lesions (33), followed by coimmunoprecipitations using anti-DDB1 antibodies. Western blotting confirmed the constitutive association of endogenous DDB1 with the CRL4 complex members CUL4A and DDB2 as well as with MEKK1 in untreated cells, while, in response to DNA damage, the levels of DDB2 decreased already after 30 min, and MEKK1 levels were reduced after 4 h already in the input material (Fig. 2A). These protein decays reflect the known CRL4-mediated ubiquitin/proteasome-mediated degradation of DDB2 and p21 (32, 34), as they did not occur in the presence of the proteasome inhibitor MG132 (Fig. 2B). Similarly, the disappearance of full-length MEKK1 is due to its known caspase-mediated cleavage (22, 23) and, accordingly, was not seen in the presence of the pan-caspase inhibitor Z-VAD-FMK (Fig. 2B). A further control experiment revealed 4NQO-inducible ubiquitination of DDB2 (Fig. 2C), but the same experimental approach also showed the hitherto unknown inducible ubiquitination of DDB1 and CUL4A. To investigate the composition of the CUL4A complex, further coimmunoprecipitation experiments were performed in cells lacking individual components of the entire complex. HEK293 cells were transfected to express shRNAs against CUL4A, DDB1, DDB2, or an unspecific control and treated for 3 h with 4NQO. Coimmunoprecipitation experiments using anti-MEKK1 antibodies showed an interaction of MEKK1 with DDB2 only in the presence of DDB1 or CUL4A (Fig. 2D). In contrast, binding to DDB1 and CUL4 still occurred in the absence of the respective partner protein, consistent with a model where MEKK1 simultaneously interacts with DDB1 and CUL4 as schematically displayed in Fig. 2E.

### The kinase activity of MEKK1 triggers K63- and K48-branched autoubiquitination of the CRL4 complex.

**FIG 3:**
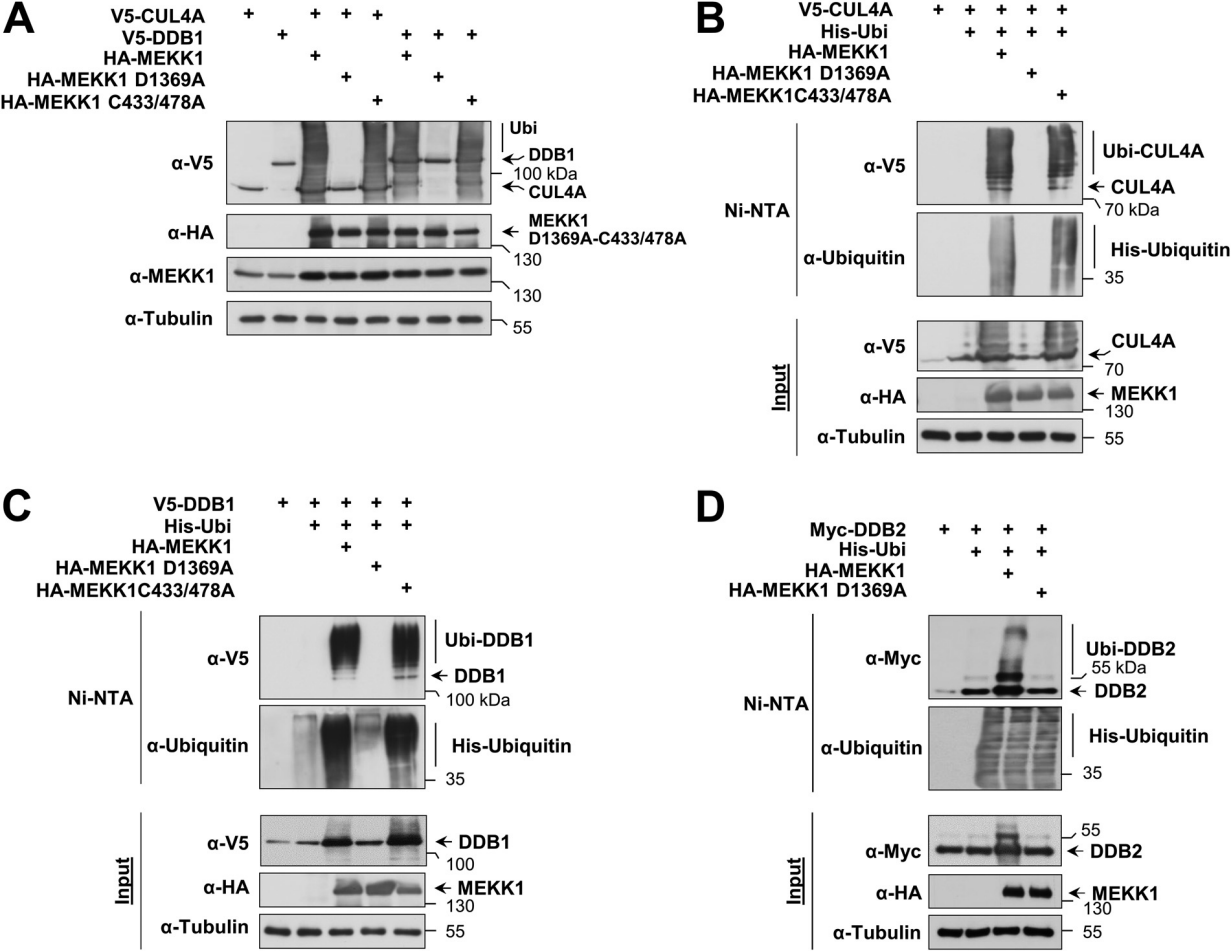
MEKK1-triggered autoubiquitination of CRL4 proteins. (A) HEK293 cells were transfected to express CRL4 complex members or MEKK1 in its WT form or variants thereof which are deficient in their function as a kinase (MEKK1 D1369A) or ubiquitin E3 ligase (MEKK1 C433/478A). Cells were lysed in 1.5× SDS sample buffer to maintain posttranslational modifications and further analyzed by Western blotting. (B) HEK293 cells were transfected to express CUL4A along with the indicated MEKK1 mutants and His-tagged ubiquitin. Ubiquitinated proteins were enriched on Ni-NTA beads under denaturing conditions, and ubiquitination of CUL4A was revealed by immunoblotting. (C and D) Experiments were performed as in panel B with the exception that ubiquitination of DDB1 and DDB2 was analyzed.

In the course of the interaction experiments, we noted that coexpression of MEKK1 caused a changed electrophoretic behavior of CRL4 complex proteins (data not shown). To test whether this depends on the enzymatic activities of MEKK1, cells were transfected to express CUL4A or DDB1 along with MEKK1 WT and mutants thereof which are defective in the kinase function (MEKK1 D1369A) or E3 ligase function (MEKK1 C433/478A). Cell lysates were prepared under denaturing conditions to preserve posttranslational modifications, followed by immunoblotting. Dependent on its kinase function, the coexpression of MEKK1 resulted in the occurrence of multiple upshifted bands for CUL4A and DDB1 representing the occurrence of posttranslational modifications (Fig. 3A). To investigate whether these upshifted forms represent ubiquitinated forms of the CRL4 complex, the experiment was repeated in the presence of coexpressed His-tagged ubiquitin, followed by enrichment of ubiquitinated proteins on nickel-nitrilotriacetic acid (Ni-NTA) columns and Western blotting. These experiments revealed the MEKK1-induced ubiquitination of CUL4A (Fig. 3B), DDB1 (Fig. 3C), and DDB2 (Fig. 3D), as shown by the enrichment of the upshifted bands representing CRL4 complex members on Ni-NTA beads.

**FIG 4:**
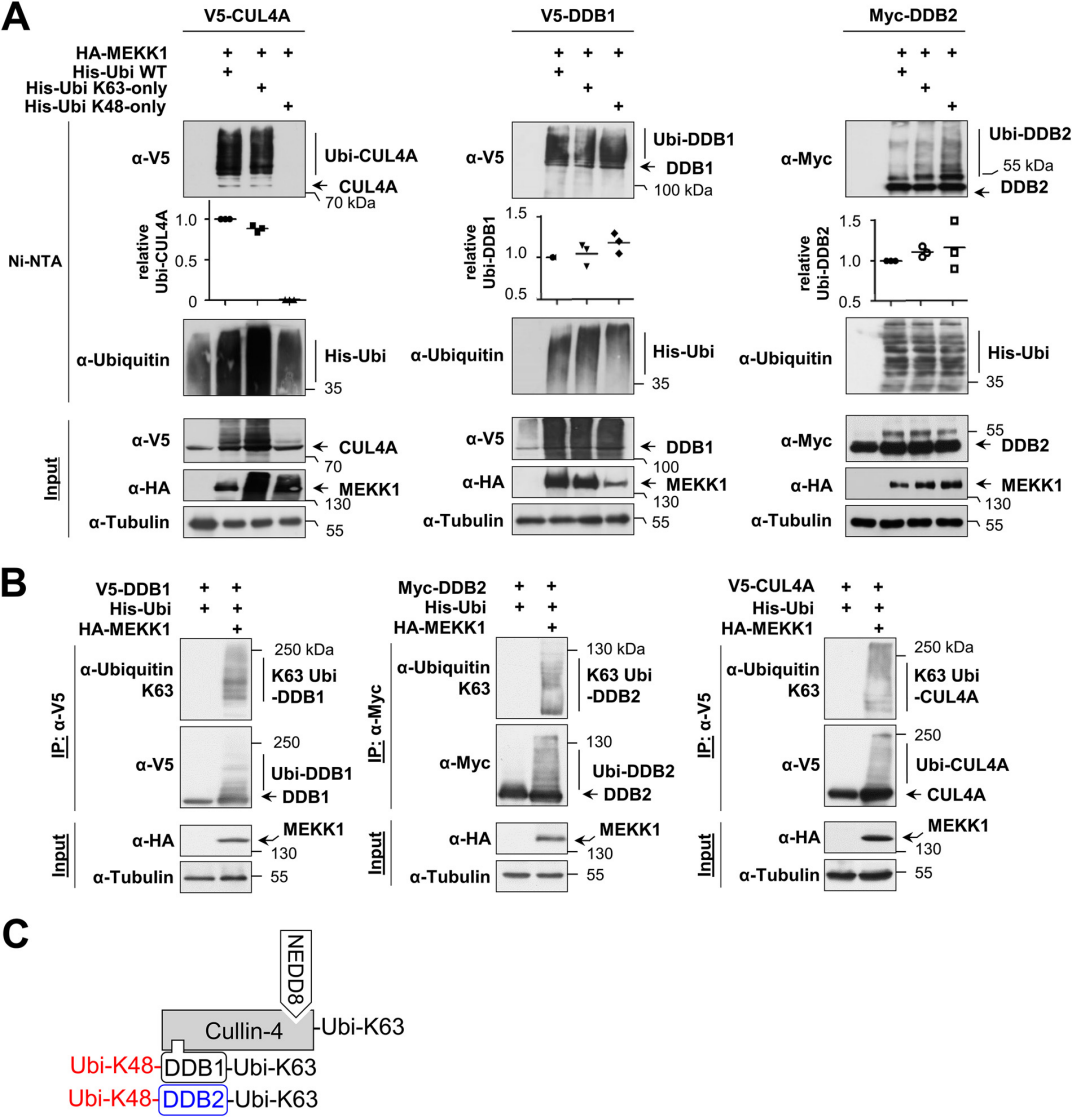
MEKK1-induced ubiquitin chains on CRL4 proteins are K48 and K63 branched. (A) CRL4 proteins and MEKK1 were expressed in HEK293 cells along with His-tagged ubiquitin that was mutated in all lysines except lysine 63 (His-ubi K63-only) or lysine 48 (His-ubi K48-only) in the presence of MG132. Following enrichment of ubiquitinated proteins on Ni-NTA beads, the ubiquitination of CUL4A (left), DDB1 (middle), and DDB2 (right) was revealed by Western blotting. Ubiquitination of the CRL4 proteins was quantified and normalized to the tubulin controls. Ubiquitination by WT ubiquitin was set to 1. Values from three independent experiments are shown, and the medians are indicated. (B) HEK293 cells were transfected to express the individual CRL4 members together with His-Ubi and MEKK1. The next day, MG132 was added overnight, and cell extracts were prepared using a buffer containing isopeptidase inhibitors. Immunoprecipitations were performed, and K63 ubiquitin chains on the CRL4 proteins were detected by Western blotting with specific antibodies. (C) Model depicting the differential MEKK1-induced ubiquitin chains on CRL4 proteins.

To identify the type(s) of ubiquitin chain branching on CRL4 complex proteins, we expressed each member of the complex along with MEKK1 and His-tagged ubiquitin or mutants thereof where all lysines except K48 (His-Ubi K48-only) or K63 (His-Ubi K63-only) were mutated to arginine. Ni-NTA purification and subsequent immunoblotting showed that MEKK1-triggered ubiquitination of CUL4A only occurred in the presence of His-Ubi K63-only but was not detected after expression of His-Ubi K48-only (Fig. 4A, left). These data suggest that MEKK1-induced CUL4A ubiquitination employs K63-linked ubiquitin chains, which are known to exert mainly nondegradative functions by enabling protein-protein interactions (35). In contrast, ubiquitination of DDB1 and DDB2 allowed MEKK1-mediated attachment of K48- and K63-branched ubiquitin chains (Fig. 4A). To substantiate our finding of MEKK1-mediated attachment of K63-branched ubiquitin chains on components of the CRL4 complex, we took advantage of the availability of specific antibodies recognizing K63-branched ubiquitin chains (36). These branching-specific antibodies detected K63 ubiquitin chains at CRL4 complex proteins immunoprecipitated from cells expressing MEKK1 (Fig. 4B). In summary, these data suggest that MEKK1 triggers signals that allow the autoubiquitination of all components of the CRL4 complex by K63-branched ubiquitin chains, while DDB1 and DDB2 can be additionally modified by K48-branched chains, as summarized in Fig. 4C.

**FIG 5:**
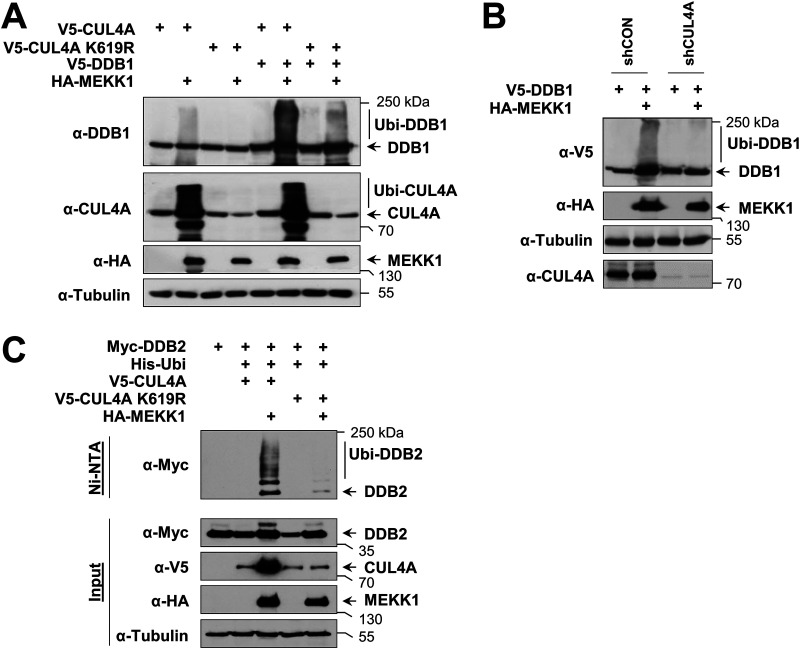
MEKK1 induces autoubiquitination of CRL4 proteins. (A) HEK293 cells were transfected with tagged DDB1 along with HA-tagged MEKK1, CUL4A, and a neddylation-deficient mutant thereof. Two days posttransfection, the cells were lysed in SDS sample buffer and analyzed for ubiquitination of CUL4A and DDB1 by immunoblotting as shown. (B) HEK293 cells were transfected with vectors encoding shRNA against CUL4A or a scrambled control together with epitope-tagged versions of DDB1 and MEKK1 as shown. Two days later, cell extracts were prepared and analyzed for protein expression and DDB1 ubiquitination by immunoblotting. (C) Cells were transfected to express DDB2 alone or in combination with CUL4A WT or CUL4A K619R together with His-ubiquitin and MEKK1 as shown. After cell lysis, ubiquitination of DDB2 was detected in Ni-NTA eluates using the anti-Myc antibody. Adequate expression of the proteins was tested in the input fraction.

A MEKK1 mutant with a defect E3 ligase activity was still able to trigger ubiquitination of the CRL4 complex (Fig. 3), raising the possibility that CRL4 modification proceeds by autoubiquitination or alternatively by another unknown E3 ligase. To test the first possibility, we compared MEKK1-induced ubiquitination of CUL4A and DDB1 in cells expressing WT CUL4A and the inactive CUL4A K619R mutant. In the presence of this neddylation-defective mutant, the upshifted bands representing ubiquitinated forms of CUL4A and DDB1 were strongly reduced (Fig. 5A), suggesting the occurrence of autoubiquitination. As the residual ubiquitination of DDB1 in the presence of the CUL4A K619R mutant is probably due to the activity of the endogenous WT CUL4A, we investigated the effect of CUL4 knockdown on MEKK1-induced DDB1 ubiquitination. This ubiquitination was lost in the absence of CUL4A (Fig. 5B), supporting the occurrence of autoubiquitination. Similarly, also MEKK1-induced ubiquitination of DDB2 was not seen in the presence of CUL4A K619R (Fig. 5C), showing that MEKK1 instructs the autoubiquitination of the CRL4 complex members.

### MEKK1 contributes to DNA damage-induced degradation of p21 and DDB2 and cell survival.

**FIG 6:**
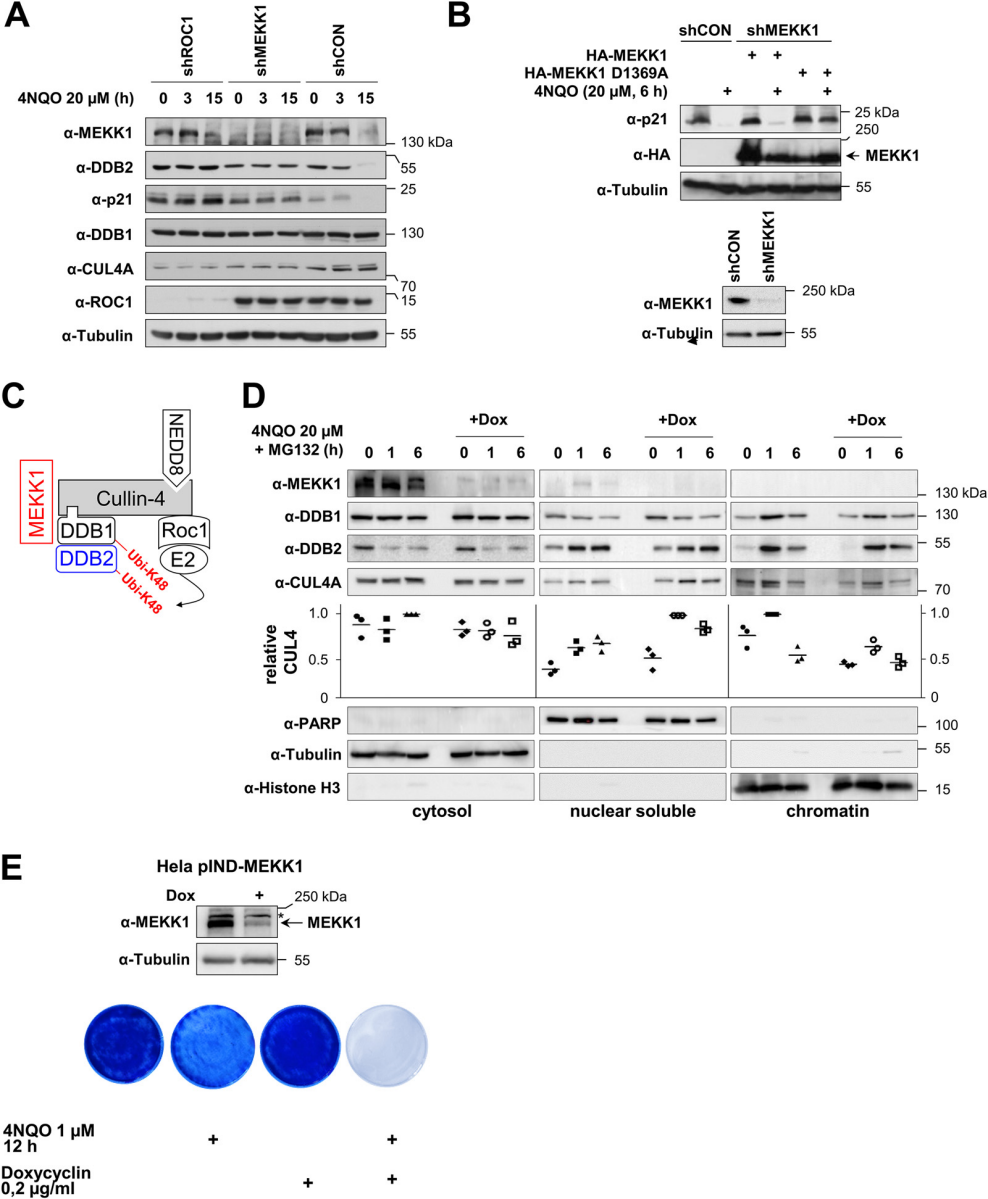
MEKK1 contributes to damage-induced degradation of p21 and DDB2 and cell survival. (A) HEK293 cells were transfected to express plasmids encoding specific shRNAs targeting ROC1, MEKK1, or a scrambled control and selected for 5 days with puromycin. Subsequently, cells were treated for various periods with 4NQO, and expression of the indicated proteins was revealed by immunoblotting. (B) HEK293 cells were transfected to express MEKK1-specific or control shRNAs and selected for 5 days with puromycin. Subsequently, cells were reseeded and transfected as shown with shRNA-resistant forms of MEKK1 and its mutants, followed by 4NQO treatment and Western blotting to detect p21 degradation (top) and MEKK1 knockdown (bottom). (C) Schematic summary of the results. (D) HeLa cells allowing the Dox-inducible expression of miR30-based shRNA for MEKK1 were generated (HeLa pIND-MEKK1) and treated for 4 days with Dox. Cells were then exposed for the indicated periods to 4NQO (to induce DNA damage) and MG132 (to prevent degradation of DDBs), followed by fractionation of cells into cytosolic, nuclear, and chromatin fractions. These fractions were analyzed for the kinetics of inducible CUL4A and DDB2 import into the nucleus and chromatin. The purity of the fractions was controlled by blotting for tubulin (cytosol), PARP (nuclear soluble), and histone H3 (chromatin). The relative levels of CUL4A in the various fractions was quantified using the ChemiDoc imaging system, and relative protein amounts were normalized to the marker protein of the respective fraction. Maximal expression in each fraction was arbitrarily set to 1; the median values are indicated. (E) HeLa pIND-MEKK1 cells were treated with Dox and/or 4NQO as shown. While cells from two dishes were lysed 4 days after Dox treatment and further tested for efficient MEKK1 knockdown, dishes containing the other cells were washed, and cells were further grown to form colonies for 1 week. Cells were stained with crystal violet. Representative results are shown.

The CRL4 complex is known to mediate the DNA damage-induced ubiquitin/proteasome-dependent degradation of DDB2 and p21 in a later phase of GG-NER (32, 37). To test the contribution of MEKK1 for the function of the CRL4 complex in target protein decay, we first ensured in a test experiment that 4NQO induces the ubiquitination of p21 (see Fig. S1 in the supplemental material) and that knockdown of CUL4 or DDB1 is sufficient to inhibit the degradation of DDB2 and p21 (Fig. S2). Intriguingly, knockdown of MEKK1 prevented the 4NQO-induced decay of DDB2 and p21 (Fig. 6A), revealing an important contribution of this kinase for this degradative process. To test whether the kinase activity of MEKK1 is required for DDB2/p21 degradation, cells were transfected with an shRNA targeting the endogenous kinase and retransfected to express shRNA-resistant forms of MEKK1 WT and the kinase-inactive MEKK1 D1369A mutant. 4NQO-induced degradation of p21 only occurred in the presence of MEKK1 WT, while cells expressing the kinase-inactive mutant showed no p21 decay (Fig. 6B), as also summarized schematically in Fig. 6C. We then generated a cell line allowing the doxycycline (Dox)-inducible expression of a miR30-based MEKK1-specific shRNA (HeLa pIND-MEKK1 cells). As DNA damage promotes the translocation of the CRL4 complex to the chromatin fraction where it binds to damaged DNA (38), it was interesting to investigate whether (i) also MEKK1 undergoes this inducible translocation, and (ii) whether MEKK1 affects this process. To address these questions, HeLa pIND-MEKK1 cells were treated with Dox to mediate MEKK1 downregulation and exposed for various periods to 4NQO, followed by cell fractionation and detection of CRL4 complex proteins. Immunoblotting revealed the inducible enrichment of DDB1, DDB2, and also CUL4 in the nuclear soluble and chromatin fraction, while MEKK1 stayed entirely in the cytosol (Fig. 6D). While downregulation of MEKK1 did not significantly affect the nuclear translocation of DDB1 and DDB2, it had a mild but consistently observed effect on the 4NQO-induced chromatin translocation of CUL4 (Fig. 6D). One hour post-cell stimulation, the absence of MEKK1 caused an increase of CUL4 in the nuclear fraction and a decrease in the chromatin fraction, as seen by Western blotting and its quantitative analysis. These data suggest that MEKK1 is exclusively associated with the cytoplasmic CRL4 complex, and, consistent with this result, we found no role of MEKK1 for the nuclear process of NER (Fig. S3). To detect a potential role of MEKK1 on 4NQO-induced cell death, HeLa pIND-MEKK1 cells were treated with 4NQO either alone or in combination with Dox to trigger MEKK1 downregulation. Surviving cells were grown into colonies and stained with crystal violet. These experiments showed that 4NQO-induced cell death was increased in the absence of MEKK1, as seen by crystal violet staining of surviving cells (Fig. 6E) and their quantitative analysis (Fig. S4), suggesting a prosurvival function of this kinase in the DNA damage response.

### K63-branched ubiquitination contributes to DNA damage-induced DDB2/p21 decay, cell cycle regulation, and cell survival.

**FIG 7:**
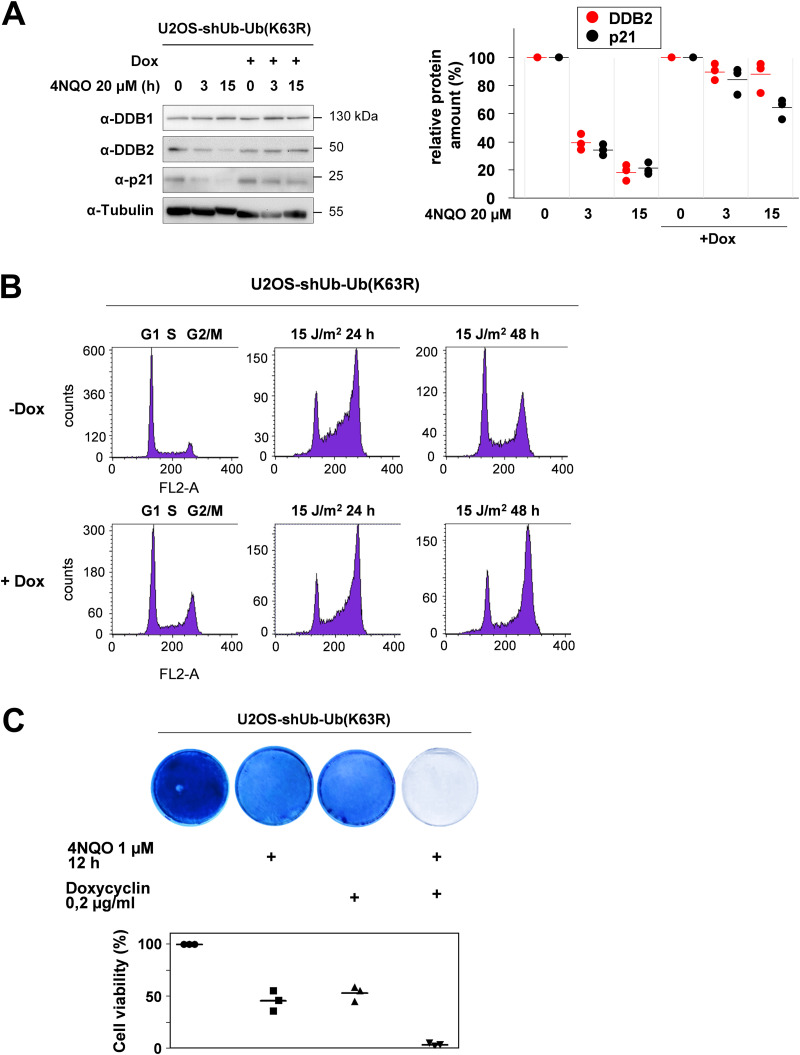
K63 ubiquitin chains contribute to DNA damage-induced decay of p21 and DDB2 and the regulation of cell cycle reentry and survival. (A) U2OS-shUb-Ub (K63R) cells were treated for 2 days with Dox, followed by the addition of 4NQO as shown. (A, left) Cell extracts prepared to monitor the decay of p21 and DDB2 by immunoblotting. (A, right) Quantification of protein expression from three independent experiments. Maximal expression in untreated cells was set to 1. Medians are indicated. (B) U2OS-shUb-Ub (K63R) cells were treated for 2 days with vehicle (−Dox) or Dox, followed by irradiation with UV and addition of fresh medium. The cell cycle distribution was analyzed in controls and 1 or 2 days after irradiation by fluorescence-activated cell sorter (FACS). A representative result is shown, and the cell cycle phases are indicated. (C) U2OS-shUb-Ub (K63R) cells were treated for 2 days with Dox and then treated for 12 h with 4NQO as shown. After exchange of the medium, the surviving cells were further grown to colonies that were stained with crystal violet. The bottom part shows a quantitative analysis of these experiments using the crystal violet assay kit. Staining of the untreated cells was set to 100%, and median values from three independent experiments are displayed.

While MEKK1-triggered addition of K48-branched ubiquitin chains to the target protein(s) leads to their proteasomal degradation, the function of MEKK1-triggered K63-linked ubiquitin chains are not known. To test the function of K63 ubiquitin chains, we employed a U2OS osteosarcoma cell line that allows Dox-regulated silencing of endogenous ubiquitin by shRNAs and simultaneous inducible expression of an shRNA-resistant ubiquitin variant harboring a K63R mutation (see Fig. S5) (39). These U2OS-shUb-Ub (K63R) cells were used to test the impact of K63 ubiquitin chains on 4NQO-induced degradation of DDB2 and p21. Cells were treated with Dox for 2 days to enable expression of K63R ubiquitin, followed by 4NQO treatment for various periods. 4NQO-induced degradation of DDB2 and p21 was largely inhibited by interference with K63-branched ubiquitin, as seen in immunoblots and their quantitative analysis (Fig. 7A). It was then interesting to investigate a possible contribution of this ubiquitin linkage for the process of DNA damage-regulated cell cycle arrest and reentry. In control cells, a pulse of UV radiation caused a transient accumulation of cells at G_2_/M, followed by normalization of cell cycle distribution after 2 days. In contrast, U2OS cells expressing K63R-mutated ubiquitin chains remained arrested at G_2_/M (Fig. 7B), indicating a relevant role of these ubiquitin chains for dynamic cell cycle regulation after DNA damage. Also, cell survival after induction of 4NQO-triggered cell death required the ability to form K63-branched chains, as seen by crystal violet staining of surviving cells and their quantitative analysis (Fig. 7C). In summary, these results reveal a relevant function of MEKK1-triggered CRL4-mediated K48- and K63-linked ubiquitin chains for the decay of p21 and DDB2 and cell cycle regulation.

## DISCUSSION

Cells contain hundreds of different CRL complexes with individual substrate receptors differing in abundance by up to 200-fold (11). These dynamic complexes are controlled by neddylation, exchange factors, and also by further posttranslational modifications and the association with additional proteins such as MEKK1, as revealed in this study. We assume that MEKK1 is not a stoichiometric interactor of cullin-4 and is, rather, associated with a subfraction of CRL4 complexes in the cytosol. It would be very interesting to know whether the activating function of MEKK1 on CRL4 function relies on the direct phosphorylation of the CRL4 complex or, alternatively, on a scaffolding function of this large kinase. The regulatory function of MEKK1 may also involve its ability to interact with CSN6 (constitutive photomorphogenesis 9 [COP9] signalosome 6), a component of the COP9 signalosome with a function in cullin deneddylation (40), but on the other hand, we could not observe robust effects of MEKK1 on the neddylation status of cullin-4 (data not shown). Interestingly, MEKK1 also inducibly binds to the ubiquitin E3 ligase Itch in response to T cell activation, leading to a phosphorylation-dependent conformational change and elevated catalytic activity of Itch (41, 42). Together, these findings raise the possibility that MEKK1 acts as a toggle to convert rapid phosphorylation cascades into ubiquitination events either by its intrinsic ability to function as an E3 ligase or by mediating indirect effects on other E3 ligases such as Itch or CRL4. The CRL4 complex can mediate different types of ubiquitination, including monoubiquitination of histones (38), K48-linked ubiquitination of its degradation targets (43), and nonproteolytic K29-linked ubiquitination (44). To our knowledge, CRL4-mediated K63 ubiquitination has thus far only been observed using *in vitro* assays for BECLIN 1 as a substrate (45), while our data suggest that MEKK1 can instruct CRL4 to mediate K63 ubiquitination *in vivo*. Neither MEKK1 nor K63-linked ubiquitination contributes to the direct process of NER (see Fig. S6 in the supplemental material) but, rather, function during later phases in the process where nucleosome-released DDB2 is degraded to enable DNA damage handover to drive further steps of GG-NER (46). We suggest that MEKK1-activated CRL4 does not lead to the degradation of only proteins such as DDB2 and p21, which play multiple roles in the regulation of DNA damage, cell proliferation, and survival (47, 48), but also of further proteins that need to be identified in the future.

**FIG 8:**
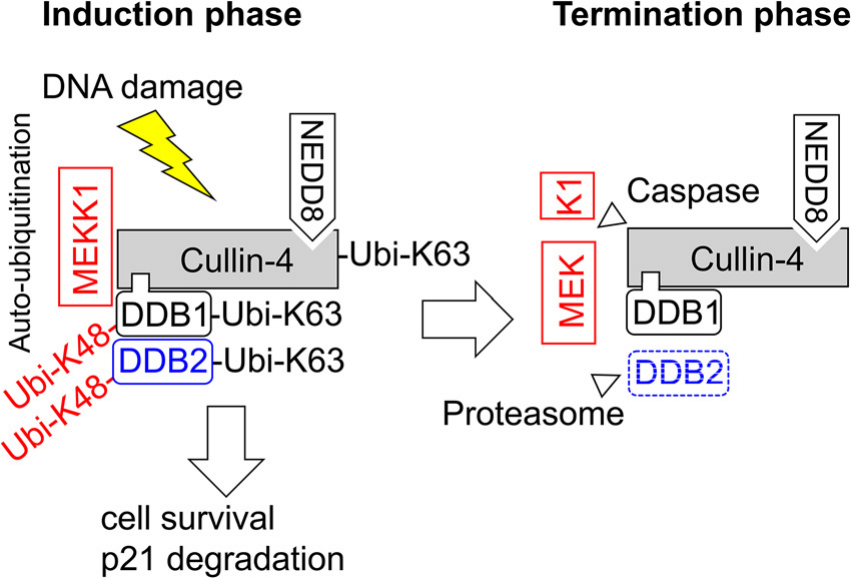
Schematic model depicting the results. During the initiation phase of the DNA damage response, MEKK1 enables the autoubiquitination of the CRL4 complex and the destruction of its substrate proteins. During the termination phase, the caspase-mediated cleavage of MEKK1 disables its CRL4 association.

How can K63 chains contribute to the decay of DDB2 and p21 and cell cycle regulation? As schematically depicted in Fig. 8, MEKK1 enables, during the initiation phase of the DNA damage response, the autoubiquitination of CRL complexes by K63-branched ubiquitin chains, while this process is terminated later on by caspase-mediated cleavage of this kinase. It is well established that K63 ubiquitination does not lead to direct proteasomal degradation, which, rather, employs K48- and K11-branched ubiquitin chains (35, 49). It is therefore plausible that K63 chains are not involved in the direct process of protein degradation but, rather, enable the transient association of protein complexes, which, in turn, instruct downstream processes leading to protein decay (50). Formally, we cannot rule out a direct contribution of K63-branched chains for protein decay, as short K63 ubiquitin chains can seed the formation of mixed K63/K48-branched ubiquitin chains together with ubiquitin escaping the shRNA-mediated knockdown in our cell system. Such K63/K48 hybrid chains allow, for example, the Itch-mediated proteasomal degradation of the proapoptotic regulator thioredoxin-interacting protein (TXNIP) (51).

The newly identified function of MEKK1 as a regulator of CRL4 might have pathophysiological implications, as loss-of-function mutations of *Map3k1* are frequently found in tumors of breast, prostate, and colon (52, 53). These tumors will not only be affected by changes in MEKK1-dependent kinase signaling pathways (53) but could also display changes in the degradation of proteins with a role in DNA damage and repair, thus possibly contributing to genomic instability frequently observed in cancer cells (54). In addition, MEKK1 inactivation may affect the ability of CRL4 to degrade tumor suppressors or oncoprotein (5) or reprogram CRL4 for degradation of neosubstrates in response to therapeutic thalidomide derivatives.

## MATERIALS AND METHODS

### Cell culture and transfection.

U2OS-shUb-Ub (K63R) cells (39), human embryonic kidney 293 (HEK293) cells, and HeLa cells and their derivatives were cultivated in Dulbecco’s modified Eagle medium (DMEM) supplemented with 10% (vol/vol) fetal calf serum, 100 U/ml penicillin, and 100 μg/ml streptomycin. Cells were transfected by mixing linear polyethylenimine (2.5 μg per μg of DNA) in serum- and antibiotic-free DMEM with DNA, and liposomes were formed upon incubation at room temperature for 30 min. Meanwhile, the medium of the cells to be transfected was replaced by antibiotic-free DMEM. The transfection suspension was then added dropwise to the cells, gently mixed, and incubated for 6 h in an incubator at 37°C in a humidified atmosphere containing 5% (vol/vol) CO_2_. Afterward, the medium was exchanged to fresh complete DMEM. Transfected cells were lysed after 2 days or (in the case of MEKK1 knockdown experiments) further grown in the presence of puromycin (1 μg/ml) to ensure complete knockdown of the kinase and elimination of untransfected cells. Knockdown of endogenous ubiquitin mRNA (RSP27A) and reexpression of mutated ubiquitin transcripts were validated by reverse transcriptase quantitative PCR (RT-qPCR).

### Cell lysis and fractionation.

Cells were lysed under different conditions. Lysis under denaturing conditions was performed by adding 1.5× SDS sample buffer to the washed cell pellets, which were then resuspended. Extracts were sonicated two times for 20 s with a Branson sonifier to shear the genomic DNA. After boiling the samples for 5 min, the lysates were analyzed by Western blotting. For subcellular fractionation experiments, pelleted cells were lysed in 200 μl buffer A (10 mM HEPES [pH 7.0], 10 mM KCl, 0.1 mM EDTA, 0.1 mM EGTA, 1 mM β-mercaptoethanol, and 0.5 mM phenylmethylsulfonyl fluoride [PMSF]) and incubated for 10 min on ice. Igepal CA-630 (Sigma-Aldrich) was added to a final concentration of 0.25% (vol/vol), and samples were briefly vortexed and centrifuged for 10 s at 16,000 × *g*. The supernatants representing the cytoplasmic fractions were collected in fresh tubes and mixed with 5× SDS sample buffer. The remaining pellets were washed twice in 1 ml buffer A and cleared by centrifugation at 13,000 rpm for 10 s. For preparation of nuclear extracts, pellets were resuspended in 100 μl buffer B (20 mM HEPES [pH 7.9], 400 mM NaCl, 1 mM EDTA, 1 mM EGTA, 1 mM β-mercaptoethanol, and 0.5 mM PMSF) and incubated on ice for 20 min while being agitated several times. After centrifugation at 13,000 rpm for 10 min, supernatants corresponding to the nuclear fraction were collected in fresh tubes. The remaining pellets representing the chromatin fractions were washed twice in buffer B and then resuspended in 100 μl 1× SDS sample buffer, boiled, and sheared three times for 20 s with a sonifier. The purity of the cellular fractions was confirmed by Western blotting, detecting tubulin (cytosolic), poly(ADP-ribose) polymerase (PARP) (nuclear), and histone H3 (chromatin) as marker proteins for the respective fraction. For native cell lysis, cell pellets were lysed on ice for 20 min in Igepal buffer (20 mM Tris-HCl [pH 7.5], 150 mM NaCl, 1% [vol/vol] Igepal CA-630, 5% [vol/vol] glycerol, and freshly added 10 mM NaF, 0.5 mM Na_3_VO_4_, 1 mM PMSF, 5 μg/ml leupeptin, and 5 μg/ml aprotinin). Lysates used for IP of ubiquitinated proteins additionally contained 10 mM *N*-ethylmaleimide and the complete protease inhibitor cocktail (Fisher Scientific, Waltham, MA USA). The lysates were cleared by centrifugation, and the supernatants were transferred into a fresh tube and either used for coimmunoprecipitation studies or mixed with sample buffer for Western blot analysis as previously described (55).

### Immunoprecipitation/coimmunoprecipitation.

Ten percent of the volume from a lysate in Igepal buffer was removed as input sample, mixed with 5× SDS sample buffer, and heated at 95°C for 5 min. The remaining lysate was precleared by the addition of 20 μl of A/G agarose bead slurry and incubated for 1 h at 4°C. Following centrifugation, the precleared lysate was transferred to a new tube, and 1 μg of the precipitating antibody or control IgG was added. After incubation at 4°C for at least 4 h, 30 μl of A/G agarose bead slurry was added, and the lysates were incubated for another 2 h at 4°C on a spinning wheel. The beads were washed five times for 5 min with Igepal buffer. After elution in 1.5× SDS sample buffer, the samples were analyzed by Western blotting.

### Ni-NTA affinity purification.

Ni-NTA was used to enrich proteins containing hexahistidine residues (His_6_) under denaturing conditions essentially as described (56). HEK293 cells were transfected with expression vectors encoding the target protein together with His-tagged ubiquitin. Cells were harvested and washed with cold 1× phosphate-buffered saline (PBS) and collected by centrifugation. After discarding the supernatant, 1 ml of Ni-NTA lysis buffer (6 M guanidine-HCl, 0.1 M Na_2_HPO_4_/NaH_2_PO_4_, 10 mM Tris, 10 mM imidazole, and 1 mM β-mercaptoethanol, pH 8.0) was added to the pellet, followed by 2 sonication steps of 20 s each. The lysates were cleared by centrifugation at 16,000 × *g* for 5 min at 4°C, the supernatants were transferred to a new tube, and 10% (vol/vol) of the total volume was stored as the input fraction. The rest of the supernatant was mixed with 50 μl equilibrated Ni-NTA–agarose and incubated for 2 h at room temperature on a rotating device. The beads were washed once in Ni-NTA lysis buffer and then in consecutive steps once in urea wash buffer 1 (8 M urea, 0.1 M Na_2_HPO_4_/NaH_2_PO_4_, 10 mM Tris base, 10 mM imidazole, and 1 mM β-mercaptoethanol, pH 8.0) and twice in urea wash buffer 2 (8 M urea, 0.1 M Na_2_HPO_4_/NaH_2_PO_4_, 10 mM Tris, 10 mM imidazole, 1 mM β-mercaptoethanol, and 0.1% [vol/vol] Triton X-100, pH 6.3). The bound proteins were eluted by boiling the beads for 4 min in 50 μl 2× SDS sample buffer containing 200 mM imidazole. The eluates were transferred into a fresh tube and analyzed by Western blotting.

### Colony formation assay and crystal violet staining.

Equal numbers of cells were grown and treated as specified in the figure legends. Afterward, the medium was removed, and cells were washed with PBS and further grown in complete DMEM medium until colonies were visible. Then, the cells were washed with PBS, fixed with ice-cold methanol for 10 min, and stained with 0.5% (wt/vol) crystal violet in 25% (vol/vol) methanol for 10 min. The excess dye was removed by rinsing the cells twice with distilled water, and the plates were photographed. Results from crystal violet staining were quantified using the crystal violet assay kit (Abcam; catalog no. ab232855).

### Fluorescence-activated cell sorter analysis.

Cell cycle distribution was analyzed in a FACSCalibur (BD Biosciences, San Jose, CA, USA) flow cytometer using propidium iodide (PI) staining. For the measurement, cells were grown on 6-cm dishes, and cells were gently harvested upon detachment with TrypLE Express (Fisher Scientific, Waltham, MA USA). Cells were washed twice with PBS, and the pellet was resuspended in 300 μl PBS. To fix the cells, 1 ml 70% (vol/vol) cold ethanol was added dropwise under constant agitation to avoid the formation of cell clumps, and the cells were incubated at 4°C for at least 1 h. After that, cells were washed again twice with PBS and resuspended in 500 μl PI staining solution (20 μg/ml propidium iodide, 200 μg/ml RNase A, and 0.1% [vol/vol] Triton X-100 in PBS), followed by incubation for 15 min at 37°C in the dark. To stop the reaction, samples were put on ice. The PI signal was detected in the red fluorescence channel (FL2). Data were recorded and evaluated with the BD CellQuest Pro software.

### DNA repair assays.

After treatment of cells, genomic DNA was extracted using a purification kit (Macherey-Nagel, Düren, Germany). Enzyme-linked immunosorbent assays (ELISAs) detecting cyclobutane pyrimidine dimers were done as described by the manufacturer (Cosmo Bio, Carlsbad, USA; catalog no. NMDND001). Briefly, a 96-well microtiter plate was first precoated at 37°C overnight with a 0.003% (wt/vol) protamine sulfate solution and then coated with denatured genomic DNA (200 ng/well) at 37°C overnight. The immobilized DNA was washed 5 times with PBS-T (PBS containing 0.05% [vol/vol] Tween 20), and the plates were blocked with 2% (vol/vol) fetal bovine serum (FBS) in PBS at 37°C for 30 min. After incubation with the first antibody, TDM-2 (1:3,000 in PBS), and several washing steps, peroxidase-streptavidin-coupled secondary antibody was added for 30 min at 37°C. After five washes with PBS-T, plates were washed with citrate-phosphate buffer, and the substrate solution was added for 30 min at 37°C. The enzyme reaction was stopped with 2 M H_2_SO_4_, and the absorbance at 450 nm was determined with GloMax device (Promega, Walldorf, Germany).

### Plasmids and antibodies.

Plasmids and antibodies are listed in Table S1 in the supplemental material.
