# Supplementary material for: MEKK1-Dependent Activation of the CRL4 Complex Is Important for DNA Damage-Induced Degradation of p21 and DDB2 and Cell Survival
Source: Mol Cell Biol. 2021 Sep 24;41(10):e00081-21. doi: 10.1128/MCB.00081-21 (PMC8462458; doi:10.1128/MCB.00081-21)
Supplement: Supplemental file 1 — Fig. S1 to S6 and Table S1. Download MCB.00081-21-s0001.pdf, PDF file, 0.8 MB [file mcb.00081-21-s0001.pdf]

Supplementary Figures

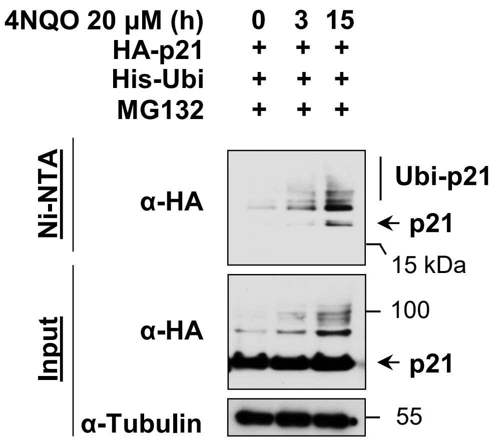

Suppl. Fig. S1. Equal numbers of HEK293 cells were transfected with vectors encoding HA-p21 and His-tagged ubiquitin. The next day, MG132 (10 µM) was added to prevent protein decay and cells were treated with 4NQO for the indicated periods. Cells were lysed under denaturing conditions. While one fraction of the cells was analyzed for expression of p21, another fraction was used for enrichment of ubiquitinated proteins on Ni-NTA beads, followed by detection of p21 ubiquitination, the position of the ubiquitinated p21 forms is indicated.

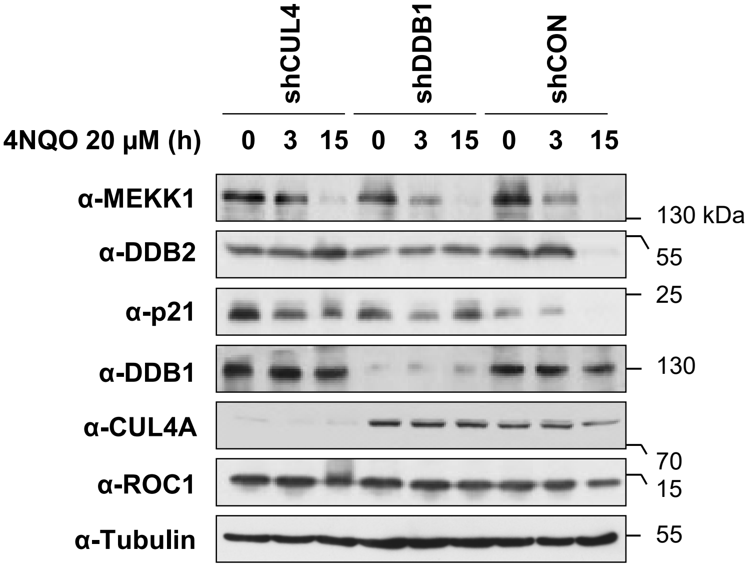

Suppl. Fig. S2. HEK293 cells were transfected to express plasmids encoding specific shRNAs targeting CUL4, DDB1 or a scrambled control and selected for 2 days with puromycin. Subsequently cells were treated for various periods with 4NQO and expression of the indicated proteins was revealed by immunoblotting.

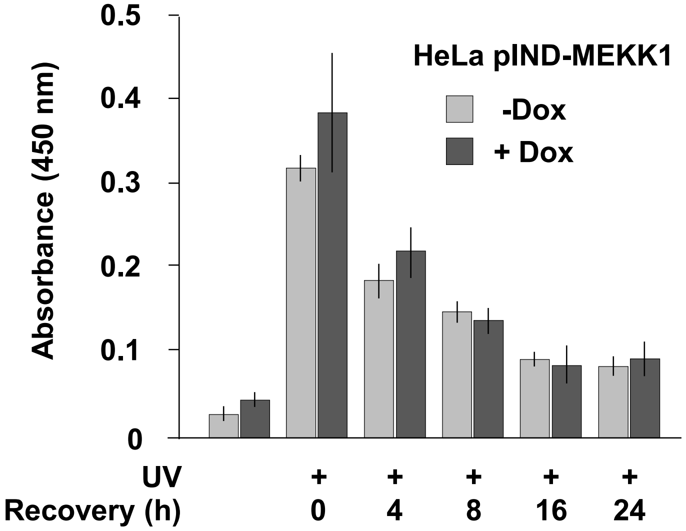

Suppl. Fig. S3. Hela pIND-MEKK1 cells were treated with Dox (0,2 µg/ml) or DMSO as vehicle for 4 days. Following a pulse of UV radiation (15 J/m^2), cells were further grown for different periods in DMEM medium to allow the occurrence of DNA repair. Genomic DNA was extracted and the amount of thymidine dimers was detected using ELISA assays. Error bars show standard deviations from three independent experiments.

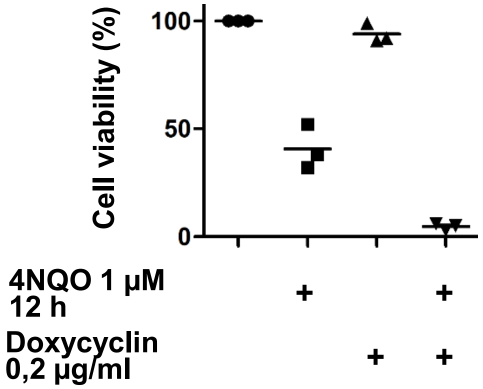

Suppl. Fig. S4. Hela pIND-MEKK1 cells were treated with Dox and/or 4NQO as shown, washed and cells were further grown to form colonies for one week. Cells were stained with crystal violet and solubilized, followed by determination of optical density at 595 nm. Density of untreated cells was set to 100%, median values from three independent experiments are displayed.

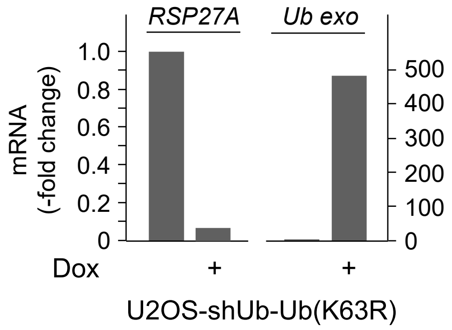

Suppl. Fig. S5. U2OS-shUb-Ub (K63R) cells were treated for three days with Dox as shown. Suppression of endogenous ubiquitin mRNA (RSP27A) and re-expression of mutated ubiquitin transcripts (exogenous ubiquitin; Ub exo) was determined by RT-qPCR as described (PMID: 27315556), a representative experiment is shown.

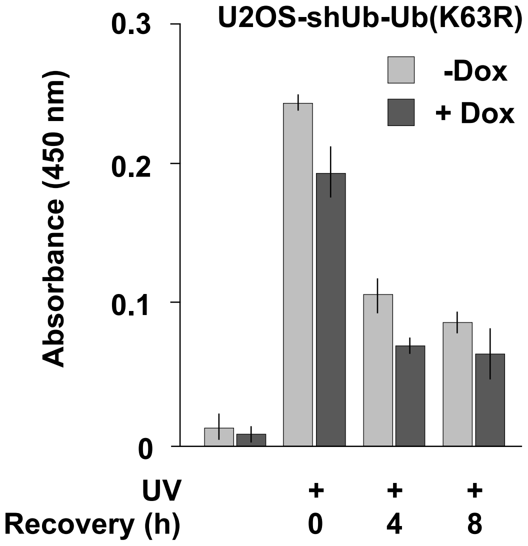

Suppl. Fig. S6. U2OS-shUb-Ub (K63R) cells were treated for two days with Dox or vehicle (DMSO). Following a pulse of UV radiation (15 J/m^2), cells were further grown for different periods in DMEM medium to allow the occurrence of DNA repair. Genomic DNA was extracted and the amount of thymidine dimers was detected using ELISA assays. Error bars show standard deviations from three independent experiments.

Stekman et al. supplementary table 1

Primary Antibodies

Primary antibody	Species	Dilution	Supplier
HA	rat mAb (3F10)	WB: 1:2000	Sigma
V5	mouse mAb (SV5-PK1)	WB: 1:2000	Thermo Fisher
Myc	mouse mAb (9E10)	WB: 1:2000	Santa Cruz Biotechnology
Flag	mouse mAb (M2)	WB: 1:5000	Sigma
DCAF7	rabbit pAb	WB: 1:2000	Dr. A.V. Skurat, Indiana
Tubulin	mouse mAb [E7]	WB: 1:1000	Sigma
Ubiquitin	mouse mAb (P4D1)	WB: 1:1000	Cell Signaling
Ubiquitin K63	rabbit mAb (D7A11)	WB: 1:500	Cell Signaling
p21	mouse mAb (F5)	WB: 1:1000	Santa Cruz Biotechnology
ROC1	rabbit mAb (D3J5I)	WB: 1:1000	Cell Signaling
PARP	mouse mAb (C2-10)	WB: 1:1000	Santa Cruz Biotechnology
Histone H3	rabbit pAb	WB: 1:5000	Abcam
MEKK1	rabbit pAb (C-22)	WB: 1:1000	Santa Cruz Biotechnology
DDB1	mouse mAb (2B12D1)	WB: 1:1000	Invitrogen
CUL4A	rabbit pAb (ab72548)	WB: 1:1000	Abcam
CUL4A	rabbit mAb (EPR3198)	WB: 1:1000	Abcam
DDB2	mouse mAb (2246C4a)	WB: 1:1000	Santa Cruz Biotechnology
DDB2	rabbit pAb (H-127)	WB: 1:1000	Santa Cruz Biotechnology
Cyclobutane pyrimidine dimers	mouse mAb (TDM-2)	ELISA: 1:3000	Cosmo Bio

Mouse IgG control	sc-2025	WB: 1:1000	Santa Cruz Biotechnology
Rat IgG control	sc-2026	WB: 1:1000	Santa Cruz Biotechnology

Secondary Antibodies

Secondary antibody	conjugated with	Dilution	Supplier
GARabbit	HRP	WB: 1:5000	Dianova
GAM	HRP	WB: 1:5000	Dianova
GARat	HRP	WB: 1:5000	Dianova

Plasmids

Plasmid	Origin	Reference
shCON (pSUPERpuro scramble)	Dr. M.L. Schmitz	PMID: 20940704
shDCAF7 (pSUPERpuro-Han11)	Dr. M.L. Schmitz	PMID: 20940704
shMEKK1 (pSUPERpuro-MEKK1)	Dr. M.L. Schmitz	this study
shROC1 (pSUPERpuro-ROC1)	Dr. R. Bernards, Amsterdam	PMID: 19723642
shDDB1 (pRETROsuper-puro-DDB1)	Dr. R. Bernards, Amsterdam	PMID: 19723642
shDDB2 (pRS-DDB2)	Dr. M.L. Schmitz	this study
pINDUCER-MEKK1	Dr. M.L. Schmitz	this study
V5-CUL4A and mutants	Dr. Y. Xiong, Chapel Hill (Addgene)	PMID: 15655366

V5-DDB1	Dr. Y. Xiong, Chapel Hill (Addgene)	PMID: 15655366
HA-p21	Dr. E. Shibata, Charlottesville	PMID: 21628527
HA-MEKK1 (and mutants)	Dr. M.H. Cobb, Dallas	PMID: 12228228
Flag-MEKK1	Dr. M.L. Schmitz	this study
Flag-ΔMEKK1	Dr. T. Maniatis, New York	PMID: 9008162
Flag-Cullin 1-7	Dr. E. Burstein, Dallas	PMID: 17183367
Myc-DDB2	Dr. Y. Xiong, Chapel Hill (Addgene)	PMID: 15448697
His-Ubi	Dr. R. Baer, New York	PMID: 14671306
His-Ubi K48 only	Dr. R. Baer, New York	PMID: 14671306
His-Ubi K63 only	Dr. R. Baer, New York	PMID: 14671306
pIND-MEKK1	Dr. M.L. Schmitz	this study
